# Fucoxanthin inhibits tumour‐related lymphangiogenesis and growth of breast cancer

**DOI:** 10.1111/jcmm.14151

**Published:** 2019-01-16

**Authors:** Jia Wang, Yanhong Ma, Jingshi Yang, Lu Jin, Zixiang Gao, Lingyun Xue, Lin Hou, Linlin Sui, Jing Liu, Xiangyang Zou

**Affiliations:** ^1^ Regenerative Medicine Center, The First Affiliated Hospital of Dalian Medical University Dalian China; ^2^ Department of Biotechnology Dalian Medical University Dalian China; ^3^ Department of Critical Care Medicine The First Affiliated Hospital of Dalian Medical University Dalian China; ^4^ INNOBIO Corporation Limited Dalian China; ^5^ College of Life Sciences Liaoning Normal University Dalian China

**Keywords:** fucoxanthin, lymphatic metastasis, MDA‐MB‐231 cells, tumour‐induced lymphangiogenesis

## Abstract

Tumour lymphangiogenesis plays an important role in promoting the growth and lymphatic metastasis of tumours. The process is associated with cell proliferation, migration and tube‐like structure formation in lymphatic endothelial cells (LEC), but no antilymphangiogenic agent is currently used in clinical practice. Fucoxanthin is a material found in brown algae that holds promise in the context of drug development. Fucoxanthin is a carotenoid with variety of pharmacological functions, including antitumour and anti‐inflammatory effects. The ability of fucoxanthin to inhibit lymphangiogenesis remains unclear. The results of experiments performed as part of this study show that fucoxanthin, extracted from *Undaria pinnatifida* (Wakame), inhibits proliferation, migration and formation of tube‐like structures in human LEC (HLEC). In this study, fucoxanthin also suppressed the malignant phenotype in human breast cancer MDA‐MB‐231 cells and decreased tumour‐induced lymphangiogenesis when used in combination with a conditional medium culture system. Fucoxanthin significantly decreased levels of vascular endothelial growth factor (VEGF)‐C, VEGF receptor‐3, nuclear factor kappa B, phospho‐Akt and phospho‐PI3K in HLEC. Fucoxanthin also decreased micro‐lymphatic vascular density (micro‐LVD) in a MDA‐MB‐231 nude mouse model of breast cancer. These findings suggest that fucoxanthin inhibits tumour‐induced lymphangiogenesis in vitro and in vivo, highlighting its potential use as an antilymphangiogenic agent for antitumour metastatic comprehensive therapy in patients with breast cancer.

## 1 INTRODUCTION

**Figure 1:**
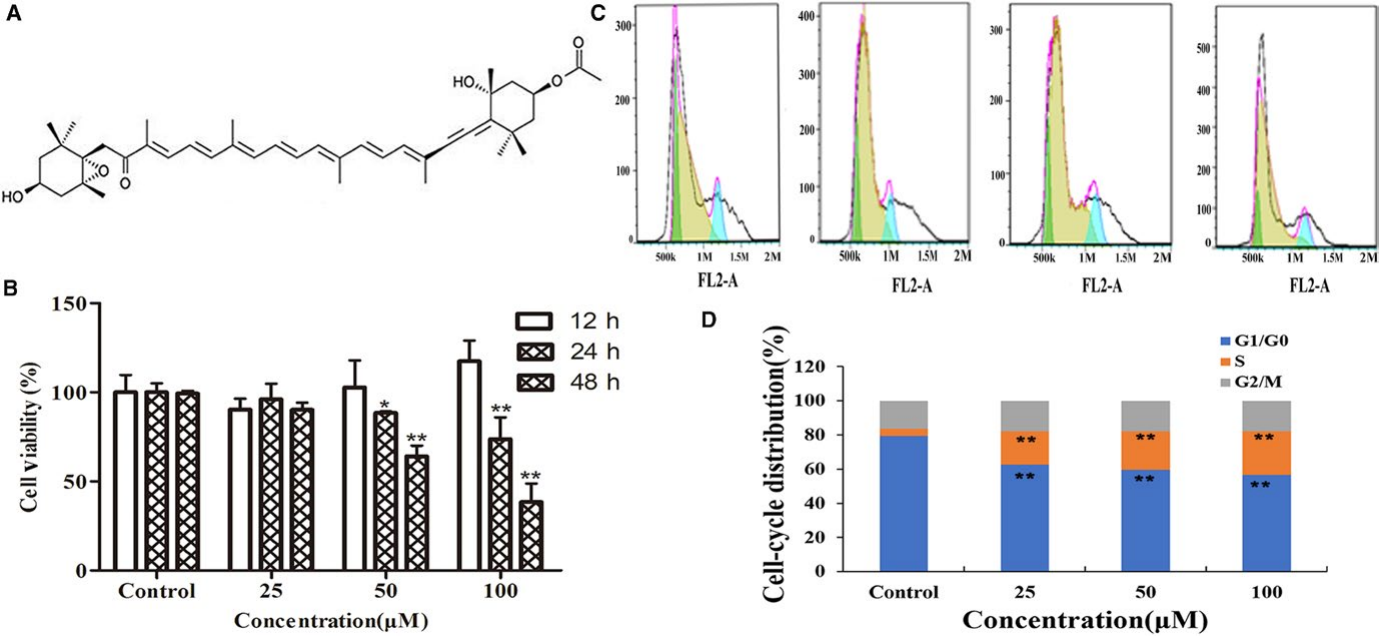
Effect of fucoxanthin on viability and cell cycle distribution in human lymphatic endothelial cells. A, Chemical structure of fucoxanthin. B, Cell viability after 12, 24 or 48 h in culture. C, Flow cytometry histograms and (D) cell cycle distribution as assessed via flow cytometry. After 24 h, fucoxanthin treatment arrested cells in the S phase and significantly decreased length of the G0/G1 phase. Values are mean ± SD. *P < 0.05 and **P < 0.01 vs untreated control (one‐way ANOVA)

Fucoxanthin is an orange, non‐pro‐vitamin A carotenoid found in brown algae such as *Undaria pinnatifida* (Wakame) and *Eisenia bicyclis* (Arame) 1. The structures of fucoxanthin (3′‐acetoxy‐5,6‐epoxy‐3,5′‐dihydroxy‐6′,7′‐didehyro‐5,6,7,8,5′,6′‐hexahydro‐β,β‐carotene‐8‐one) is shown in Figure 1A. Fucoxanthin has recently been shown to exert important biological effects, including antitumour, antioxidant and antidiabetic activity 2. Previous studies in human umbilical vein endothelial cells (HUVEC) have shown that fucoxanthin exerts an antiangiogenic effect that contributes to the prevention of cancer^3^. Fucoxanthin prevents the proliferation of tumour cells through classical pathways involved in metastasis and the cell cycle, including the PI3K/Akt and nuclear factor kappa B (NF‐κB) pathways^4^. Although fucoxanthin has been found to play an important role in human health, specific effects on tumour lymphatic metastasis remain to be elucidated. Here, we explore the effects of fucoxanthin on lymphangiogenesis induced by MDA‐MB‐231 breast cancer cells.

Lymphatic metastasis, a process in which tumour cells migrate from primary tumours to lymph nodes via lymphatic vessels, facilitates metastasis of malignant tumours. Lymphatic vessel formation is observed in diverse types of tumour tissue, including tumour vessels, tumour cells, tumour‐associated fibroblasts and inflammatory cells. Lymphangiogenesis involves the proliferation and migration of lymphatic endothelial cells (LEC) in lymphatic vessels surrounding the tumour; this process is promoted by the secretion of lymphangiogenic factors from inflammatory and tumour cells. Lymphangiogenic factors such as vascular endothelial growth factor (VEGF)‐C, which binds to VEGF receptor‐3 (VEGFR‐3), increase the ability of lymphatic vessels to invade tumours 6. Expression of VEGF‐C was found to correlate with metastasis in human cancer 7, 8. In animal models, inhibition of lymphangiogenesis through blockade of VEGF‐C or VEGFR‐3 has been shown to prevent lymphatic metastasis. Ongoing studies are investigating the role of VEGFR‐3 and VEGF‐C/‐D in tumour lymphangiogenesis, using soluble VEGFR‐3 or antibodies to VEGFR‐3 to inhibit tumour metastasis and lymphangiogenesis 9.

Nuclear factor kappa B is another molecule that plays an important role in lymphangiogenesis. Nuclear factor kappa B is a transcription element located in the cytoplasm. Before release is triggered by a stimulus, NF‐κB remains bound in an inert NF‐kappa‐B inhibitor (IκB) complex. On stimulation, a nuclear localization signal regulates transport of the activated NF‐κB complex into the nucleus to induce related gene expression, in particular, VEGF‐C, Cyclin D1, matrix metalloproteinase‐2 (MMP‐2) and matrix metalloproteinase‐9 (MMP‐9). Nuclear factor kappa B may be activated by numerous growth factors, cytokines and receptor families associated with tumour necrosis factor. Nuclear factor kappa B is also triggered by other signalling pathways, such as PI3K/Akt and Ras/MAPK, to induce antiapoptotic effects 10. PI3K/Akt signalling contributes significantly to tumour angiogenesis and carcinogenesis 11. Excitation of the PI3K/Akt/NF‐кB signalling pathway may promote tumour‐induced lymphangiogenesis, which is involved in cell proliferation and migration in LEC.

Inhibition of lymphangiogenesis represents a novel strategy for the treatment of breast cancer. Inhibition of lymphangiogenesis is expected to help control primary tumours and pre‐metastatic, tumour‐conditioned regional lymph nodes. Growth of MDA‐MB‐231 cells and tumour‐conditioned lymph nodes was mechanistically delayed by VEGFR2/3 and NRP1/2 complex formation in the presence of VEGF‐A/C 12. During the process of tumour metastasis, cellular basement membrane was prominently degraded by malignant cells, and cells spread to distant organs, resulting in the formation of secondary tumours. Various proteases, including matrix metalloproteinases (MMPs), degrade extracellular matrix (ECM) and basement membrane. Matrix metalloproteinase‐2, MMP‐9 and tissue inhibitor of metalloproteinase‐1 (TIMP‐1), the endogenous inhibitor of MMP, are highly associated with local tissue invasion, which is clearly aided by the degradation of ECM 13.

We hypothesized that ECM degradation might be of special concern because of the important role fucoxanthin plays in preventing tumour metastasis and lymphangiogenesis. So, in this study, we analyse fucoxanthin‐mediated inhibition of lymphangiogenesis and metastasis through experiments in human LEC (HLEC) and a nude mouse model of breast cancer. The fucoxanthin used was extracted from *U pinnatifida*. These experiments were designed to elucidate the mechanism underlying lymphangiogenesis to specific cellular targets.

## 2 MATERIALS AND METHODS

### 2.1 Preparation of fucoxanthin

Fucoxanthin was isolated from *U pinnatifida* and the preparation method as previously reported^14^.

### 2.2 Cell culture

Human LEC were obtained from Sciencell Research Laboratories (Carlsbad, CA; http://sciencellonline.com/). Cells were cultured in Roswell Park Memorial Institute (RPMI) 1640 medium with 15% foetal bovine serum (FBS). Human breast cancer cell line MDA‐MB‐231 was obtained from American Type Culture Collection (ATCC), where the cell lines were authenticated by short tandem repeat profiling before distribution. Cells were cultured in RPMI 1640 medium containing 10% FBS, 100 U/mL penicillin and streptomycin at 37°C in a humidified atmosphere of 5% CO_2_. Only cells at passage 3‐8 were used for experiments.

### 2.3 Cell viability

An 3‐(4,5‐dimethyl‐2‐thiazolyl)‐2,5‐diphenyl‐2‐H‐tetrazolium bromide，Thiazolyl Blue Tetrazolium Bromide (MTT) assay kit (Sigma‐Aldrich, St. Louis, MO, USA) was used to measure the effects of fucoxanthin on cell viability in vitro. Human LEC and MDA‐MB‐231 cells were cultured in 96‐well plates (1.0 × 10^4^ cells/well, in 100 μL medium) for 4 hours, then treated with fucoxanthin (25, 50, 100 μmol/L; final volume, 200 μL) for 12, 24 or 48 hours. MTT (5 mg/mL) was added to cell preparations, and plates were incubated for an additional 4 hours. Dimethyl sulfoxide (150 μL/well) was added to dissolve formazan crystals. Absorbance (*A*) was measured at 492 nm with a microplate spectrophotometer (Thermo Fisher Scientific, Carlsbad, CA). Cell viability was calculated according to the following equation:

$$\text{Cell}\;\text{viability}\left(\%\right)=\frac{\left(A_{\text{treated}}-A_{\text{blank}}\right)}{\left(A_{\text{control}}-A_{\text{blank}}\right)}\times 100\%$$

### 2.4 Flow cytometry

Human LEC (1 × 10^6^ cells/mL) were incubated for 24 hours at 37°C, then suspended in 500 μL 75% ice‐cold ethanol for >2 hours at 4°C. The resulting cell pellet was collected via centrifugation at 107 *g* for 5 minutes. Prior to incubation, 100 μL RNase A was added. Cell preparations were incubated for 30 minutes at 37°C. DNA staining was performed with propidium iodide (400 μL). Progression through the cell cycle was analysed with a FACSCalibur flow cytometer (BD Biosciences, San Jose, CA).

### 2.5 Migration assay

Transwells (6.5‐mm diameter; 8‐μm pore size) were used to measure the antimigration effect of fucoxanthin on HLEC and MDA‐MB‐231 cells. Cells (5 × 10^4^ cells/well) were plated on the upper Transwell chamber and treated with various concentrations of fucoxanthin in serum‐free medium; the lower chamber contained fresh medium without fucoxanthin. After 24 hours in culture, cotton swabs were used to remove non‐migrating cells on the upper surface of the filter. Cells on the lower surface that had passed through the membrane were fixed with 70% ethanol, then stained with 0.1% crystal violet for 8 minutes. Images of five fields were obtained with a microscope (Olympus, Tokyo, Japan). The number of migrated cells in each image was counted. Values averaged across five fields were recorded.

### 2.6 Cell invasion

MDA‐MB‐231 cells treated with fucoxanthin (25, 50, 100 μmol/L) for 24 hours were incubated in serum‐free medium. For invasion assays, 1 × 10^5^ cells were plated to the top chambers of Transwell inserts coated with Matrigel (Sigma‐Aldrich). Then, 500 mL medium containing 10% FBS was added as a chemoattractant to the lower chambers. After incubation for 24 hours at 37°C, cells on the upper surface of the insert were removed by swabbing. Cells that had migrated were fixed with 70% ethanol for 10 minutes and stained with 0.1% crystal violet for 8 minutes. Microscopic images were used to count the number of cells that had migrated. Counts for five randomly selected fields were averaged.

### 2.7 Tube formation

A Matrigel‐based assay was used to measure tube formation, in order to analyse the effect of fucoxanthin on formation of lymphatic‐like structures in vitro. Briefly, HLEC (1 × 10^4^ cells/well) were plated in 96‐well plates that had been pre‐incubated with Matrigel at 37°C. Various concentrations of fucoxanthin were added before cells had attached to the Matrigel. Cell preparations were then incubated for 24 hours. An inverse microscope (NOVEL, Nanjing, China) was used to obtain images ≥3 fields per well. The rate of tube formation was calculated as follows:

$$\text{Formation}\;\text{rate}\left(\%\right)=\frac{\text{Tube}\;{\text{quantity}}_{\text{treated}}}{\text{Tube}\;{\text{quantity}}_{\text{control}}}$$

### 2.8 Enzyme‐linked Immunosorbent Assay

MDA‐MB‐231 cells were incubated with fucoxanthin (25, 50, 100 μmol/L) for 24. The supernatant collected was analysed by ELISA. Each 96‐well plate was coated with polyclonal anti‐VEGF‐C (1:1500). Staining was measured at 450 nm on a Multiskan Ascent photometer (Thermo Fisher Scientific). Relative amounts of phospho‐Flk‐1/Flt‐4 were determined with a phospho‐Flk‐1/Flt‐4 cell‐based colorimetric ELISA kit (ImmunoWay Biotech, Plano, TX).

### 2.9 Immunohistochemistry

To stain the cytoskeleton, cells were fixed in 3.7% methylaldehyde for 10 minutes, washed with PBS containing 0.1% TritonX‐100 for 5 minutes, and then stained for 20 minutes at room temperature with green‐fluorescent phalloidin (KeyGEN Biotech, Nanjing, China). Cell nuclei were identified by counterstaining with 4′,6‐diamidino‐2‐phenylindole. After three washes, cells were examined under a fluorescent microscope (Leica, Solms, Germany).

### 2.10 Western blot

To examine protein expression levels in HLEC after treatment with fucoxanthin, cells were treated with fucoxanthin for 24 hours, then lysed in RIPA lysis buffer (Thermo scientific, Waltham, MA，USA). Equivalent amounts of protein (40 μg) were measured using the Bradford method and resolved by 12% SDS‐PAGE gels, which were electrotransferred onto nitrocellulose membranes. Membranes were incubated overnight at 4°C with relevant primary and peroxidase‐conjugated secondary antibodies. Protein bands were detected with an ECL system (Bio‐Rad Laboratories, Hercules, CA). The following primary antibodies were used: human anti‐p‐PI3K, p‐Akt, NF‐κB, VEGF‐C, VEGFR‐3, MMP‐2, MMP‐9, TIMP‐1(1:500) and anti‐GAPDH (the latter as internal control).

### 2.11 Quantitative real‐time PCR (Real‐time qPCR)

Cells treated with fucoxanthin were cultured for 24 hours. TRIzol reagent (Invitrogen, Carlsbad, CA) was used to extract total RNA. Reverse‐transcription used an oligo(dT) primer and Murine Leukemia Virus reverse transcriptase (Takara, Dalian, China) to obtained cDNA *GAPDH* was used as internal control. The following primers were used: *GAPDH*, 5′‐TGGCACCCAGCACAATGAA‐3′ and 5′‐CTAAGTCATAGTCCGCCTAGAAG CA‐3′; *VEGF‐C*, 5′‐ATTAGACGTTCCCTGCCAGC‐3′ and 5′‐TCCAGCTCCTTGTTTGGTCC‐3′; *VEGFR‐3*, 5′‐ATTCCCCATGACCCCAACGA‐3′ and 5′‐GTAAAA CACCTGGCCTCCTCG‐3′. Real‐time qPCR was performed in triplicate for every sample in a parallel design, using the SYBR^®^ Premix ExTaq^™^ (Takara) and the Takara detection system TaKaRa TP800 (Dalian, China), according to the manufacturer's protocol. Subsequently, the genes expression data were analysed by Thermal Cycler Dice Real Time system software (Takara), and quantified by the comparative cycle threshold (Ct) method (2^−ΔΔCt^ method), based on the Ct values for among VEGFC, VEGFR3 and GAPDH to calculate the relative fold increase.

### 2.12 Conditional culture

Briefly, MDA‐MB‐231 cells were treated with fucoxanthin (25, 50, 100 μmol/L) for 24 hours and cultured with serum‐free medium for 24 hours. Supernatant was centrifuged and collected. Human LEC were cultured with 40% conditional medium for assays of tube formation.

### 2.13 Lymphangiogenesis in vivo

The protocol was approved by the Animal Care and Ethics Committee of Dalian Medical University. Balb/c nu/nu mice (female mice, 5‐6 weeks old, approximately 18 g) were purchased from Dalian Medical University Experimental Animal Center. Animals were housed under specific pathogen‐free conditions. The tumour model was established in 18 mice through inoculation with MDA‐MB‐231 cells (1 × 10^7^ cells, 100 μL), which were injected subcutaneously into the right armpit. After 5 days, mice were randomly divided into control and experimental groups. Experimental groups were treated with fucoxanthin; normal saline (NS) was used for control treatment. Fucoxanthin (100, 500 μmol/L; 100 μL per mouse) or NS (100 μL) was injected at the tumour periphery every day, for 26 days. Tumour length and width were measured with calipers every 4 days. All mice were euthanized on day 26, at which point tumours were isolated and weighed. Tumour volumes were calculated as *V *= *ab* ^2^/2 (*a*, tumour length; *b*, tumour width). Tumours were fixed in 10% neutral formaldehyde, embedded in paraffin and sectioned. Lymphatic vascular density in tumour tissue was quantified by immunofluorescence. Animal studies were performed according to the appropriate ethical criteria and approved by the Committee for the Care and Use of Animal Subjects at Dalin Medical University.

### 2.14 Statistical analysis

Data are expressed as mean ± SD. Significant differences were evaluated by one‐way ANOVA using spss and Prism 5 (Version 5.04, GraphPad Software, Inc, La Jolla, CA). Statistical significance was defined as *P < *0.05 and *P < *0.01. All experiments were performed at least three times for quantitative comparison.

## 3 RESULTS

### 3.1 Fucoxanthin inhibits the growth and proliferation of HLEC

The effect of fucoxanthin (25, 50, 100 μmol/L) on viability and proliferation of HLEC over time (12, 24, 48 hours) was evaluated by MTT assay. Our data, as shown in Figure 1B, demonstrated that 24‐ or 48‐hour treatment with fucoxanthin inhibited cell viability. The results of flow cytometry showed that treatment of HLEC with fucoxanthin (25, 50, 100 μmol/L) for 24 hours arrested the cell cycle during S phase (Figures 1C,D).

### 3.2 Fucoxanthin inhibits tube formation in HLEC

**Figure 2:**
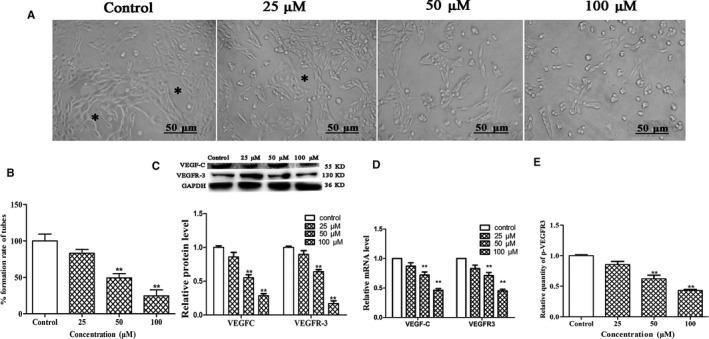
Fucoxanthin inhibits lymphatic tube formation in human lymphatic endothelial cells (HLEC). Cells were incubated for 24 h in medium with or without fucoxanthin (25, 50, 100 μM). A, Photomicrographs of HLEC tube formation (scale bar =50 μm). Complete tubular structures are marked with asterisks. B, Quantitative analysis of the rate of tube formation. C, Expression of vascular endothelial growth factor (VEGF)‐C and VEGF receptor‐3 (VEGFR‐3), as determined by Western blot. D, mRNA levels of *VEGF‐C* and *VEGFR‐3*, as determined by Real‐time qPCR. E, p‐VEGFR3 levels in fucoxanthin‐treated HLEC and controls. *P < 0.05 and **P < 0.01, compared with controls (one‐way ANOVA)

Tube formation of HLEC was assessed to investigate the antilymphangiogenic effect of fucoxanthin. Tube formation capacity was inhibited in HLEC treated with fucoxanthin for 24 hours (Figure 2A,B). mRNA and protein levels of VEGF‐C and its receptor (VEGFR‐3) decreased. The results indicated an inverse correlation between mRNA and protein levels of VEGF‐C and VEGFR‐3 and fucoxanthin concentration (Figure 2,D). Treatment with fucoxanthin decreased levels of phospho‐VEGFR3 in a concentration‐dependent manner (Figure 2E).

### 3.3 Fucoxanthin inhibits HLEC migration

**Figure 3:**
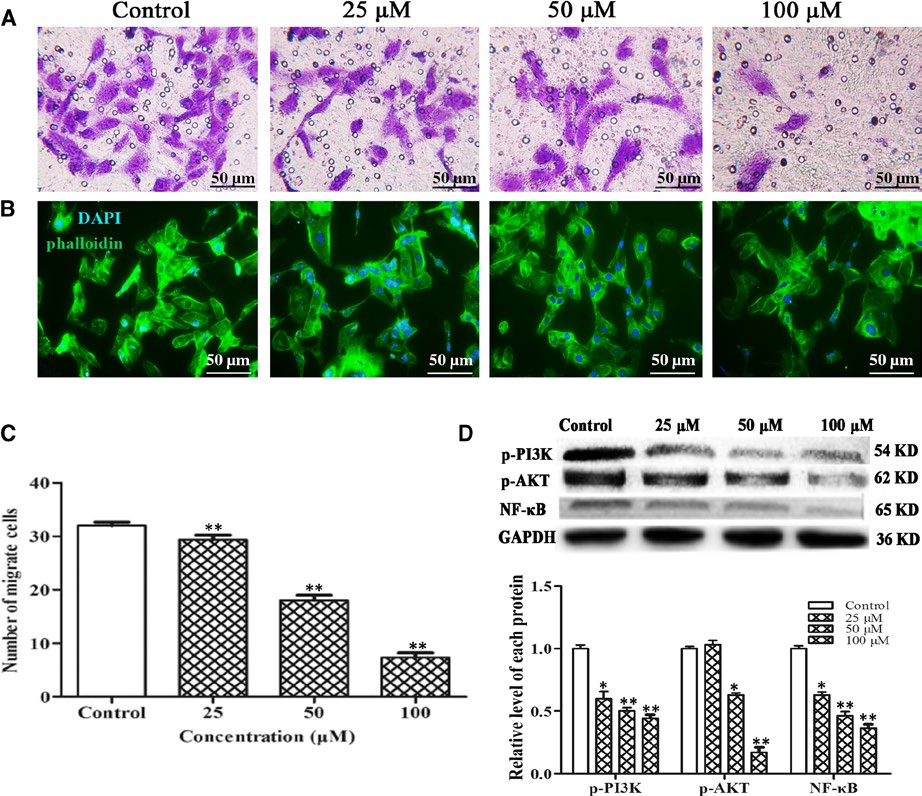
Fucoxanthin inhibits human lymphatic endothelial cells (HLEC) migration in vitro. Human lymphatic endothelial cells were incubated in medium for 24 h with or without fucoxanthin (25, 50, 100 μmol/L). A, Microscopic images (scale bar = 50 μm) and (C) number of migrated cells, as determined by Transwell assay. Averages of five fields were calculated. Data are presented as mean ± SD of three independent experiments. *P < 0.05, **P < 0.01, compared with control group (one‐way ANOVA). B, Microfilaments and nuclei were stained with phalloidin (green) and 4′,6‐diamidino‐2‐phenylindole (DAPI) (blue) respectively. D, Levels of phospho‐PI3K, phospho‐Akt and NF‐κB in HLEC treated with fucoxanthin (25, 50, 100 μmol/L), as determined by western blot. *P < 0.05, **P < 0.01, compared with controls (one‐way ANOVA)

To evaluate the effects of fucoxanthin on HLEC migration, we performed Transwell assays as well as cytoskeletal and nuclear staining. Cells treated with fucoxanthin for 24 hours migrated significantly less than control cells. This effect was concentration dependent (Figure 3A,C). Increasing concentrations of fucoxanthin affected the distribution of microfilaments within the cell, with effects on cell morphology and cell polarity (Figure 3B). These results indicate that fucoxanthin regulates the rearrangement of microfilaments to inhibit cell motility.

### 3.4 Fucoxanthin suppresses PI3K/Akt/NF‐κB signalling

In order to elucidate the mechanism underlying the inhibitory effect of fucoxanthin on lymphangiogenesis in HLEC, PI3K/Akt/NF‐κB signalling was measured by Western blot. The results indicated treatment with fucoxanthin decreased expression of phopho‐PI3K, phospho‐AKT and NF‐κB. This effect was concentration dependent (Figure 3D).

### 3.5 Fucoxanthin affects malignant phenotypes of MDA‐MB‐231 cells in vitro

**Figure 4:**
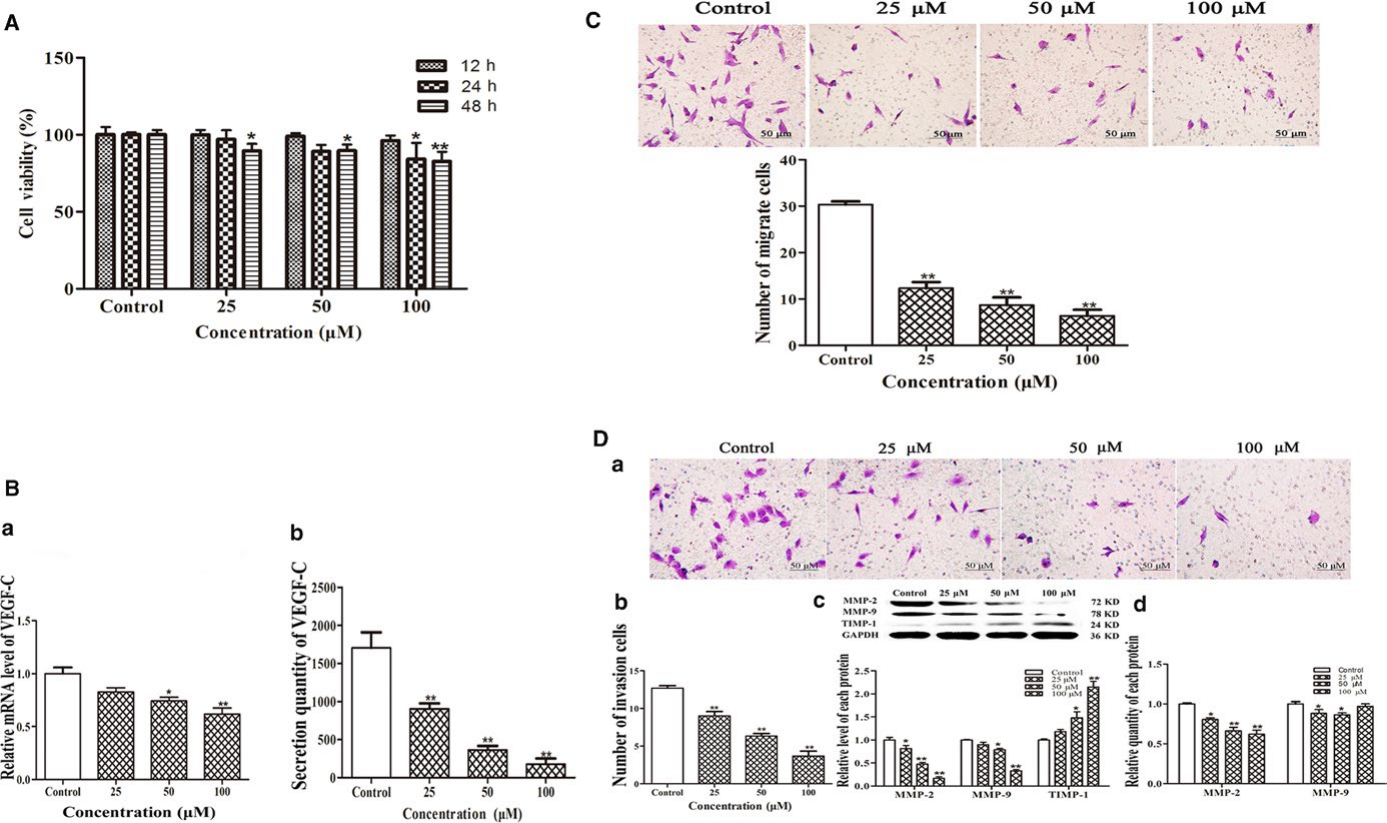
Effect of fucoxanthin on malignant phenotypes in MDA‐MB‐231 cells. Cells were treated with fucoxanthin (25, 50, 100 μmol/L) for 12‐48 h. A, Decreased cell viability in cells treated with fucoxanthin for 12‐48 h. B, Decreased mRNA and protein levels of vascular endothelial growth factor‐C after fucoxanthin treatment, as determined with real‐time qPCR and ELISA. C, Microscopic images of migration and (D) invasion in MDA‐MB‐231 cells (scale bar = 50 μm). Averages were calculated from five fields. Data are presented as mean ± SD; **P < 0.01 vs controls (one‐way ANOVA). The protein expression levels of matrix metalloproteinase‐2 (MMP‐2), matrix metalloproteinase‐9 (MMP‐9) and tissue inhibitor of metalloproteinase‐1 were measured by western blot analysis. The secretion levels of MMP‐2 and MMP‐9 were measured by ELISA.

Lymphatic metastasis requires proliferation, migration and invasion of tumour cells as well as secretion of VEGF‐C. To confirm the antimetastatic activity of fucoxanthin, we further observed the effects of fucoxanthin on malignant phenotypes (ie migration, invasion, secretion of VEGF‐C) in MDA‐MB‐231 cells with MTT and Transwell assays. As shown in Figure 4, MDA‐MB‐231 cells treated with fucoxanthin (25, 50, 100 μmol/L) for 12, 24 or 48 hours showed decreased cell viability, migration and invasion. Western blot analysis and ELISA were performed to measure expression and secretion of invasion‐associated proteins MMP‐2, MMP‐9 and TIMP‐1. The results indicated that fucoxanthin decreased the expression and secretion of MMP‐2 and MMP‐9 but increased expression and secretion of TIMP‐1(Figure 4D).

To evaluate the effect of fucoxanthin on VEGF‐C secretion, ELISA and Real‐time qPCR assays were used to measure VEGF‐C expression and secretion in MDA‐MB‐231 cells. The results showed that mRNA expression and secretion were down‐regulated in the presence of fucoxanthin (Figure 4B).

### 3.6 Fucoxanthin inhibits tumour‐induced lymphangiogenesis in vitro

**Figure 5:**
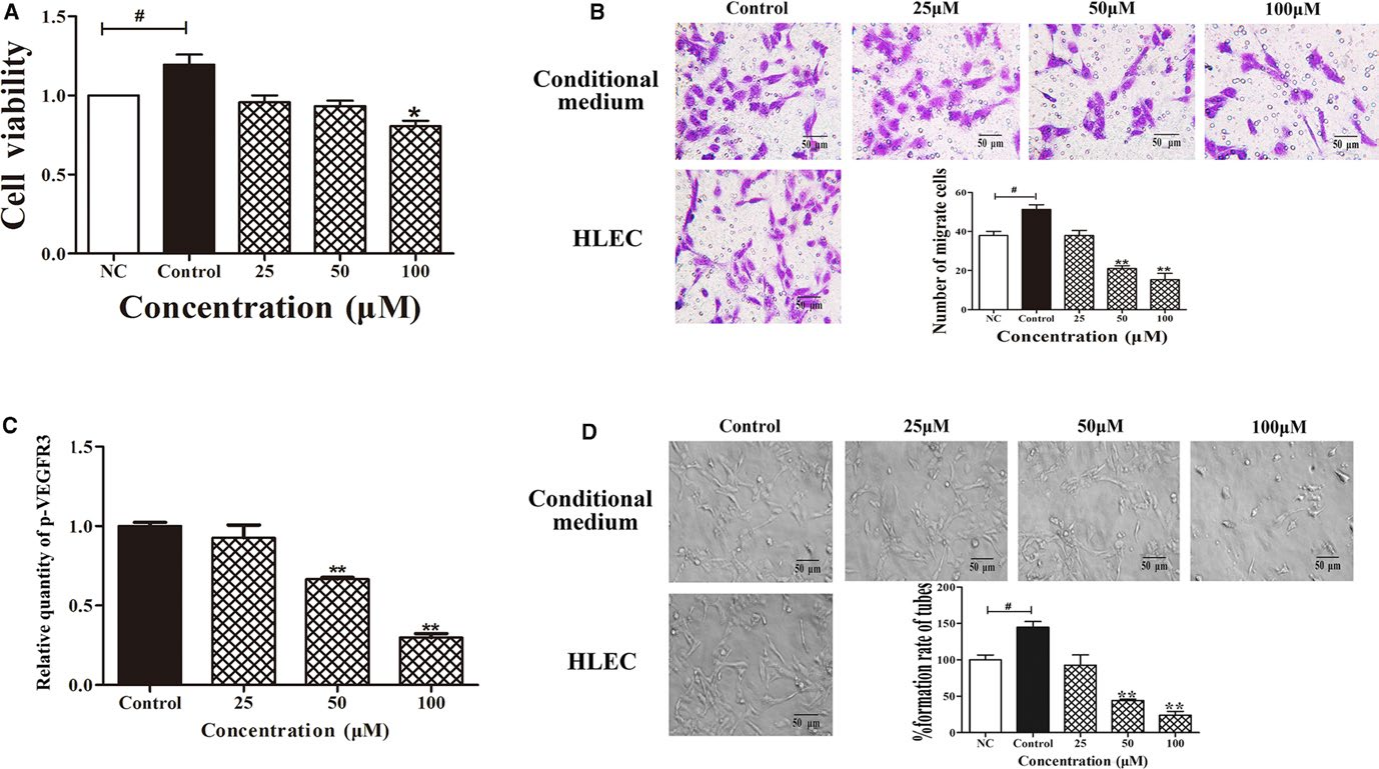
Fucoxanthin inhibits tumour‐induced lymphangiogenesis in vitro. Human lymphatic endothelial cells (HLEC) were incubated in conditional medium with or without fucoxanthin (25, 50, 100 μmol/L) for 24 h. A, Cell viability, (B) migration and (D) tube formation are decreased in HLEC. C, Results of ELISA showing fucoxanthin decreases phosphorylation of vascular endothelial growth factor receptor‐3. NC, negative control. Data are shown as mean ± SD; *P < 0.05 vs NC; *P < 0.05, **P < 0.01 vs controls (one‐way ANOVA)

Cell growth, migration and tube formation were investigated using a 24‐hour conditional culture system in order to further analyse whether fucoxanthin inhibited tumour‐induced lymphangiogenesis. Cell viability, migration and tube formation were significantly increased in the conditional culture, compared with negative controls (NC) (*P < *0.05). Treatment with fucoxanthin effectively decreased cell migration and tube formation in conditionally cultured HLEC. This effect was concentration dependent (Figure 5A,B,D). Treatment of conditionally cultured HLEC with fucoxanthin also decreased levels of phospho‐VEGFR3 (Figure 5C). These results indicate decreased levels of VEGF‐C in MDA‐MB‐231 cells.

### 3.7 Inhibition of tumour growth and lymphangiogenesis in a mouse model

**Figure 6:**
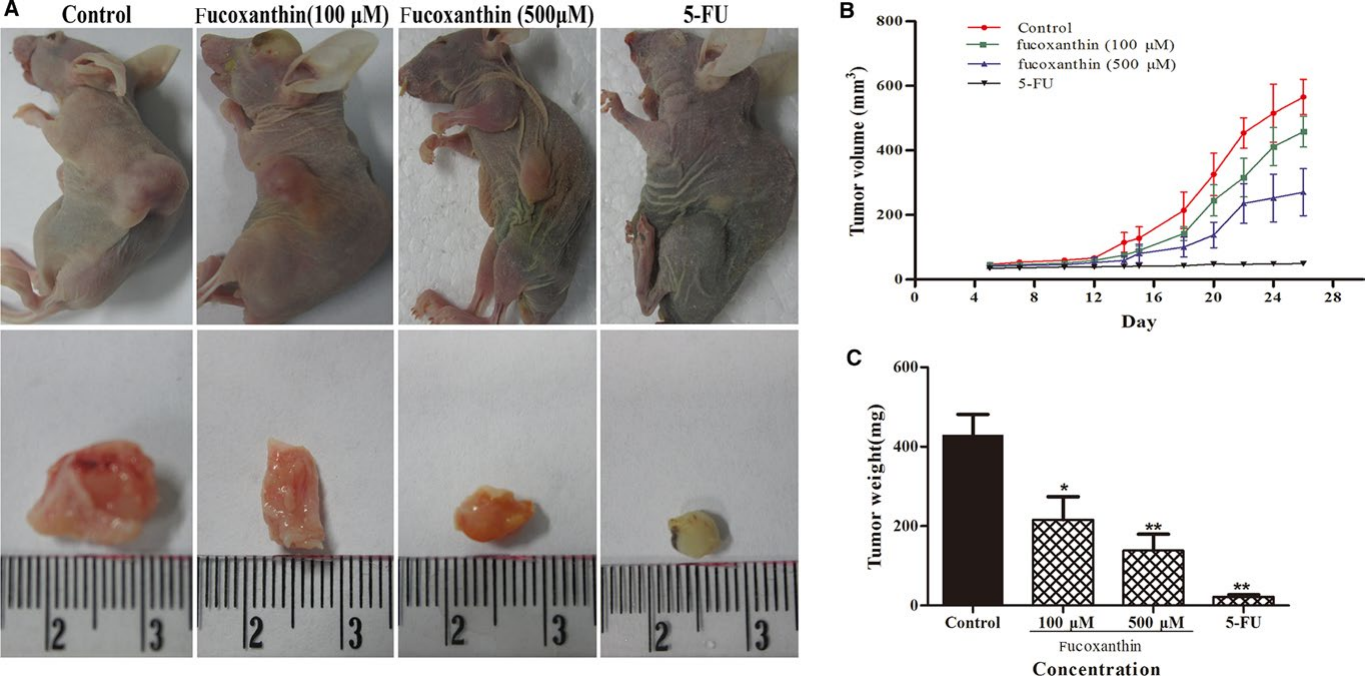
Fucoxanthin inhibited tumour growth in the MDA‐MB‐231 xenograft model of breast cancer. Female nu/nu mice were inoculated subcutaneously with MDA‐MB‐231 cells. Five days after inoculation, NS or fucoxanthin (100, 500 μmol/L; 100 μL/per mouse) was injected at the tumour's edge every day for 26 days. Tumour length and width were measured individually every 4 days. A, Representative images obtained on day 26 of tumours implanted subcutaneously. B, Tumour growth curves and (C) tumour weight. Data are presented as mean ± SD. *P < 0.05, **P < 0.01 vs controls (one‐way ANOVA)

Fucoxanthin‐mediated inhibition of tumour growth and lymphangiogenesis were investigated in a MDA‐MB‐231 breast cancer xenograft model using Balb/c nude mice. The results showed that fucoxanthin significantly decreased tumour volume (Figure 6A,B) and weight (Figure 6C).

**Figure 7:**
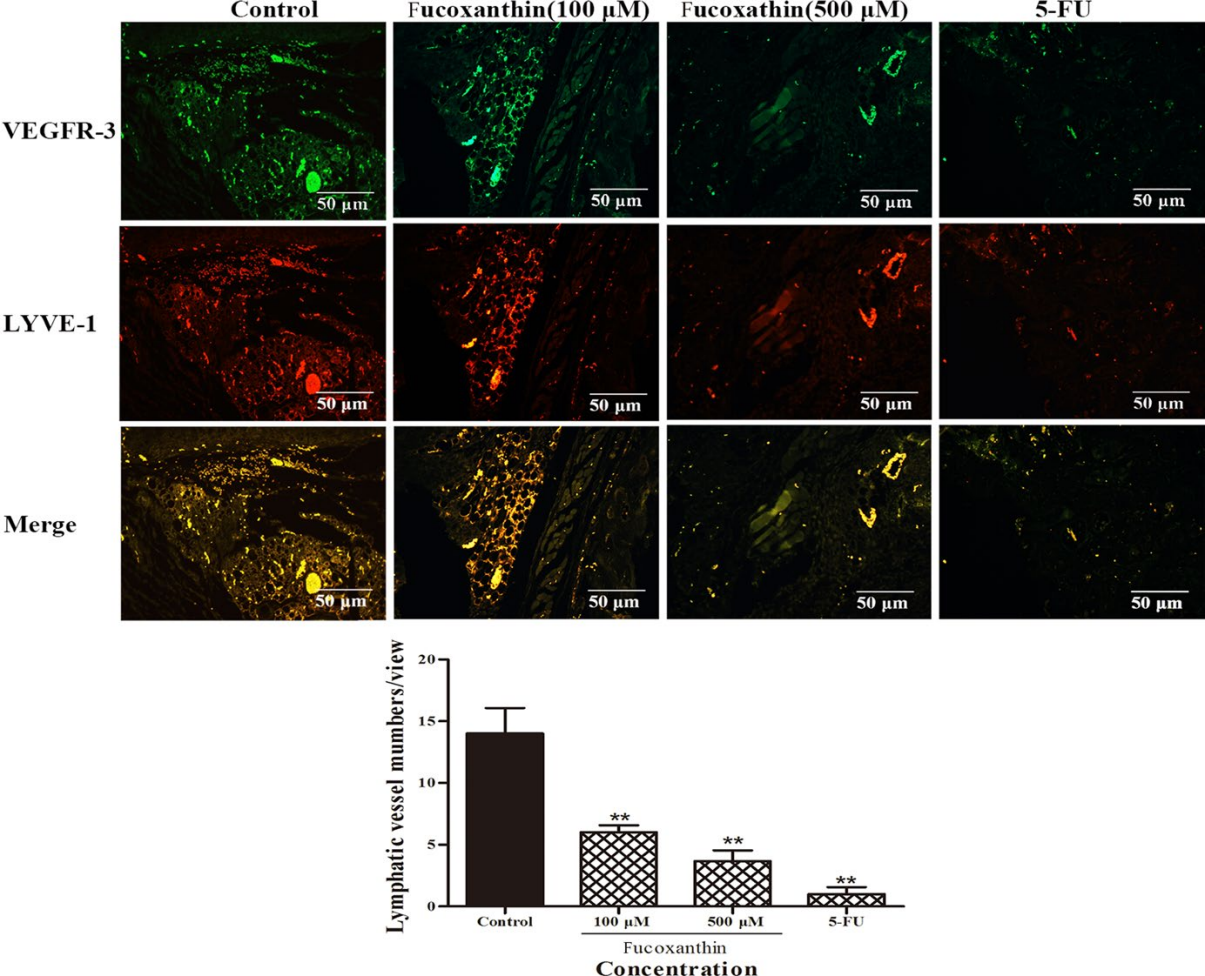
Fucoxanthin inhibits lymphangiogenesis in vivo. Top: Immunofluorescent images of tumour lymphatic vessels stained with antibodies to vascular endothelial growth factor receptor‐3 (green) and lymphatic vessel endothelial hyaluronan receptor 1 (LYVE‐1) (red), from mice inoculated with MDA‐MB‐231 cells. Bottom: Quantitative assessment of tumour lymphatic vascular density. Data are presented as mean ± SD. **P < 0.01 vs controls (one‐way ANOVA)

Tumour lymphangiogenesis was analysed using immunofluorescent staining with VEGFR‐3 and lymphatic vessel endothelial hyaluronan receptor 1 (LYVE‐1) antibodies. After treatment with fucoxanthin (100, 500 μmol/L), micro‐lymphatic vascular density (LVD) in tumour tissue significantly decreased from 14.0 ± 2.94 to 6.0 ± 0.81 and 3.66 ± 1.25 respectively (Figure 7). The results of analysis suggest that fucoxanthin inhibits tumour growth and lymphangiogenesis in a xenograft model of breast cancer.

## 4 DISCUSSION

Metastasis is a feature used for tumour classification as well as a common cause of cancer‐related death. Metastasis via lymphatic vessels is an important initial event. Tumour‐induced lymphangiogenesis in regional lymph nodes or local tumours has been associated with formation of various types of tumours, including breast cancers 15. In addition to inducing lymphangiogenesis, tumour cells at the primary site invaded existing lymphatic vessels and travelled to adjacent lymph nodes 9. Previous studies have indicated that tumour cell expression and secretion of VEGF‐C correlates with lymphangiogenesis and poor prognosis among patients with breast cancer^16^.

Fucoxanthin has potential for pharmaceutical use as an antitumour agent. Previous studies have indicated that fucoxanthin may inhibit proliferation in human cancer cells. Treatment with fucoxanthin is associated with significantly decreased expression of angiogenic factors, including VEGF 17. Low doses of fucoxanthin were shown to be effective in decreasing proliferation of MDA‐MB‐231 breast cancer cells, through inhibition of the NF‐κB pathway in a time‐dependent manner 18, 19. Although fucoxanthin acts on numerous molecular targets, studies of its effects on tumour‐induced lymphangiogenesis have failed to provide conclusive evidence for effects on HLEC.

Human LEC proliferation is closely related to the formation of a lymphatic network. The results presented above suggest that fucoxanthin inhibits HLEC proliferation by blocking the S phase of the cell cycle.

Cell migration, motility and tubulogenesis are closely related to actin‐tethered adhesion and dynamics of actin filament complexes, which comprise junctional molecules linked to the actin cytoskeleton in LEC 20, 21. We found that fucoxanthin clearly inhibited cell migration and tube formation. Treatment with fucoxanthin transformed the morphology of the HLEC cytoskeleton, supporting previous reports that fucoxanthin decreases pseudopod formation^18^.

Based on our data, we have identified the characterizing and their impact on survival，migration and invasion in MDA‐MB‐231 breast cancer cells within fucoxanthin treatment setting, along with significantly inhibitory effect accompanied by a concentration‐dependent manner. Through analysis of the literature, it appeared that expression of VEGF secreted by breast cancer cells was altered in a pro‐angiogenesis direction in breast cancers 8. As family of vascular endothelial growth factor, VEGF‐C has already been facilitated by cancer cells. Our data suggest that fucoxanthin significantly inhibited production of VEGF‐C secreted by ELISA and expression of VEGF‐C mRNA in MDA‐MB‐231 cells. MMPs are regarded as primary vital molecules that assist tumour cells during invasion or metastasis, and TIMPs modulate MMPs activation, even decreased the degradation of ECM 22. We evaluated that inhibition of relative protein and secretion of MMP‐2 and MMP‐9, and increase TIMP‐1 protein expression in MDA‐MB‐231 cells treated with fucoxanthin. Simultaneously, the results also prominently play an important role for the inhibitory action of breast cancer‐induced lymphangiogenesis treated with fucoxanthin to lay the foundation for the inhibition of lymph node metastasis of breast cancers.

Use of a conditional medium culture system allowed us to observe the interaction between two types of cells. We detected that fucoxanthin significantly inhibited cell viability, migration and tube‐like formation capacity on HLEC in conditioned medium culture system. Moreover, HLEC treated with conditioned medium also presented stronger ability of cell viability, migration and lumen structure formation. Accordingly, we verified that fucoxanthin inhibited tumour‐induced lymphangiogenesis in vitro. Vascular endothelial growth factor‐C exerts its function with binding to VEGFR‐3, causing an increase ability in proliferation and migration of HLEC and initiating the downstream signal pathways. Besides, silencing of VEGF‐C significantly inhibits lymphangiogenesis, which mostly suggested that lymphangiogenesis greatly associated with regulation of VEGF‐C. To a large extent, VEGF‐C secreted by MDA‐MB‐231 cells may promote lymphangiogenesis correlated with lymph node metastasis 23. Our results suggest that expression of VEGF‐C and VEGFR3 signalling axis was down‐regulated markedly in the protein and molecular level by high concentrations of fucoxanthin, which additionally consistent with its antilymphangiogenesis activity. We suppose that it may also laid the molecular foundation on inhibition the ability of tumour cells invading to lymphatic vessels.

Nuclear factor kappa B inhibitor could be degraded by upstream phosphorylation of Akt, allowing NF‐кB released from the cytoplasm to translocate to the nucleus and act on target genes, which all considered as the important regulatory pathways in tumour proliferation and metastasis. PI3K/Akt signalling mediates processes such as tumour angiogenesis and degradation of the ECM, as well as cell migration and adhesion 24. We studied the effects of fucoxanthin on downstream signalling by NF‐кB, p‐PI3K and p‐Akt to further explore the compound's ability to inhibit lymphangiogenesis. The results obtained showed that fucoxanthin inhibited phosphorylation of Akt and PI3K, which in turn led to the degradation of NF‐κB and decreased HLEC activation. These results indicate that fucoxanthin inhibits lymphangiogenesis by blocking NF‐кB/PI3K/Akt signalling.

Although fucoxanthin inhibited tumour‐induced lymphangiogenesis in vitro, it remained to be established whether this trend held true in vivo. We established a nude mice tumour model, breast cancer MDA‐MB‐231 cells were inoculated in nude mice, and evaluated the potential of tumour growth and lymph tube density in tumor tissue. Lymphatic vessel density is an effective indicator of response and detection of lymphangiogenesis. It is widely accepted that LYVE‐1, VEGF‐C and its receptor VEGFR‐3 are the prerequisites for the promotion of tumour lymphangiogenesis, 25, 26. LYVE‐1 and hyaluronic acid receptor homologous to CD44 (HA), which may be transported by the peripheral lymph and hyaluronic acid to induce tumour cell metastasis through lymphatic function^27^, ^28^. The results showed that the content of LVD in tumour tissues was positively correlated with the expression of LYVE‐1/VEGFR‐3 and VEGF‐C^29^. In this study, LVD was significantly lower in nude mice treated with fucoxanthin, compared to those treated with saline. The lymphatic vessels observed were also smaller and less functional. These results suggest that fucoxanthin can effectively inhibit tumour lymphangiogenesis on breast cancer cell in vivo and in vitro.

In conclusion, it may be largely demonstrated that fucoxanthin subdued the progression of lymphangiogenesis and exerted a negative role on tumour‐induced lymphangiogenesis. Fucoxanthin inhibited dramatically proliferation and migration and tube‐like structure formation of HLEC. And, activity, migration and invasion on MDA‐MB‐231 cells could be inhibited by fucoxanthin. Fucoxanthin depresses tumour‐induced lymphangiogenesis by down‐regulating secretion and mRNA level of VEGF‐C on MDA‐MB‐231cells and expression of VEGF‐C and VEGFR‐3 on HLEC, including NF‐κB degradation after inhibition of Akt and PI3K phosphorylation. Fucoxanthin is a naturally derived compound that holds promise for use as an anticancer agent.

## CONFLICT OF INTERESTS

The authors declare no conflict of interest.

## AUTHOR CONTRIBUTIONS

Jia Wang and Yanhong Ma developed the study concept and design and interpreted the data. Jingshi Yang, Lu Jin, Zixiang Gao and Lingyun Xue performed the experiments and analysed and validated the data. Lin Hou supported the data interpretation. Linlin Sui, Jing Liu and Xiangyang Zou supervised this study and wrote the manuscript.

## References

[1] D'Orazio N , Gemello E , Gammone MA , de Girolamo M , Ficoneri C , Riccioni G . Fucoxantin: a treasure from the sea. Mar Drugs. 2012;10:604‐616. 22611357 10.3390/md10030604 PMC3347018

[2] Jung HA , Ali MY , Choi RJ , Jeong HO , Chung HY , Choi JS . Kinetics and molecular docking studies of fucosterol and fucoxanthin, BACE1 inhibitors from brown algae *Undaria pinnatifida* and *Ecklonia stolonifera* . Food Chem Toxicol. 2016;89:104‐111. 26825629 10.1016/j.fct.2016.01.014

[3] Ganesan P , Matsubara K , Sugawara T , Hirata T . Marine algal carotenoids inhibit angiogenesis by down‐regulating FGF‐2‐mediated intracellular signals in vascular endothelial cells. Mol Cell Biochem. 2013;380:1‐9. 23613227 10.1007/s11010-013-1651-5

[4] Kumar SR , Hosokawa M , Miyashita K . Fucoxanthin: a marine carotenoid exerting anti‐cancer effects by affecting multiple mechanisms. Mar Drugs. 2013;11:5130‐5147. 24351910 10.3390/md11125130 PMC3877908

[5] Martin LJ . Fucoxanthin and its metabolite fucoxanthinol in cancer prevention and treatment. Mar Drugs. 2015;13:4784‐4798. 26264004 10.3390/md13084784 PMC4557004

[6] Yoshimatsu Y , Miyazaki H , Watabe T . Roles of signaling and transcriptional networks in pathological lymphangiogenesis. Adv Drug Deliv Rev. 2016;99:161‐171. 26850127 10.1016/j.addr.2016.01.020

[7] Podgrabinska S , Skobe M . Role of lymphatic vasculature in regional and distant metastases. Microvasc Res. 2014;95:46‐52. 25026412 10.1016/j.mvr.2014.07.004 PMC4446725

[8] Bender RJ , Mac GF . Expression of VEGF and semaphorin genes define subgroups of triple negative breast cancer. PLoS ONE. 2013;8:e61788. 23667446 10.1371/journal.pone.0061788 PMC3648524

[9] Dieterich LC , Detmar M . Tumor lymphangiogenesis and new drug development. Adv Drug Deliv Rev. 2016;99:148‐160. 26705849 10.1016/j.addr.2015.12.011

[10] Xi G , Hayes E , Lewis R , et al. CD133 and DNA‐PK regulate MDR1 via the PI3K‐ or Akt‐NF‐kappaB pathway in multidrug‐resistant glioblastoma cells in vitro. Oncogene. 2016;35:241‐250. 25823028 10.1038/onc.2015.78

[11] Jing Y , Liu LZ , Jiang Y , et al. Cadmium increases HIF‐1 and VEGF expression through ROS, ERK, and AKT signaling pathways and induces malignant transformation of human bronchial epithelial cells. Toxicol Sci. 2012;125:10‐19. 21984483 10.1093/toxsci/kfr256 PMC3243743

[12] Lee E , Koskimaki JE , Pandey NB , Popel AS . Inhibition of lymphangiogenesis and angiogenesis in breast tumor xenografts and lymph nodes by a peptide derived from transmembrane protein 45A. Neoplasia. 2013;15:112‐124. 23441126 10.1593/neo.121638 PMC3579314

[13] Teng H , Yang Y , Wei H , et al. Fucoidan suppresses hypoxia‐induced lymphangiogenesis and lymphatic metastasis in mouse hepatocarcinoma. Mar Drugs. 2015;13:3514‐3530. 26047481 10.3390/md13063514 PMC4483642

[14] Yu RX , Hu XM , Xu SQ , et al. Effects of fucoxanthin on proliferation and apoptosis in human gastric adenocarcinoma MGC‐803 cells via JAK/STAT signal pathway. Eur J Pharmacol. 2011;657:10‐19. 21187083 10.1016/j.ejphar.2010.12.006

[15] Li S , Li Q . Cancer stem cells, lymphangiogenesis, and lymphatic metastasis. Cancer Lett. 2015;357:438‐447. 25497008 10.1016/j.canlet.2014.12.013

[16] Cha YJ , Youk JH , Kim BG , Jung WH , Cho NH . Lymphangiogenesis in breast cancer correlates with matrix stiffness on shear‐wave elastography. Yonsei Med J. 2016;57:599‐605. 26996557 10.3349/ymj.2016.57.3.599 PMC4800347

[17] Wang J , Chen S , Xu S , et al. In vivo induction of apoptosis by fucoxanthin, a marine carotenoid, associated with down‐regulating STAT3/EGFR signaling in sarcoma 180 (S180) xenografts‐bearing mice. Mar Drugs. 2012;10:2055‐2068. 23118721 10.3390/md10092055 PMC3475273

[18] Rwigemera A , Mamelona J , Martin LJ . Comparative effects between fucoxanthinol and its precursor fucoxanthin on viability and apoptosis of breast cancer cell lines MCF‐7 and MDA‐MB‐231. Anticancer Res. 2015;35:207‐219. 25550553

[19] Rwigemera A , Mamelona J , Martin LJ . Inhibitory effects of fucoxanthinol on the viability of human breast cancer cell lines MCF‐7 and MDA‐MB‐231 are correlated with modulation of the NF‐kappaB pathway. Cell Biol Toxicol. 2014;30:157‐167. 24760606 10.1007/s10565-014-9277-2

[20] Zankov DP , Ogita H . Actin‐tethered junctional complexes in angiogenesis and lymphangiogenesis in association with vascular endothelial growth factor. Biomed Res Int. 2015;2015:314178. 25883953 10.1155/2015/314178 PMC4389985

[21] Yang Y , Gao Z , Ma Y , et al. Fucoidan inhibits lymphangiogenesis by downregulating the expression of VEGFR3 and PROX1 in human lymphatic endothelial cells. Oncotarget. 2016;7:38025‐38035. 27203545 10.18632/oncotarget.9443 PMC5122369

[22] Wang P , Liu Z , Liu X , et al. Anti‐metastasis effect of fucoidan from *Undaria pinnatifida* sporophylls in mouse hepatocarcinoma Hca‐F cells. PLoS ONE. 2014;9:e106071. 25162296 10.1371/journal.pone.0106071 PMC4146566

[23] Sun L , Duan J , Jiang Y , et al. Metastasis‐associated in colon cancer‐1 upregulates vascular endothelial growth factor‐C/D to promote lymphangiogenesis in human gastric cancer. Cancer Lett. 2015;357:242‐253. 25444928 10.1016/j.canlet.2014.11.035

[24] Yamamoto K , Ishikawa C , Katano H , Yasumoto T , Mori N . Fucoxanthin and its deacetylated product, fucoxanthinol, induce apoptosis of primary effusion lymphomas. Cancer Lett. 2011;300:225‐234. 21078541 10.1016/j.canlet.2010.10.016

[25] Yu M , Zhang H , Liu Y , et al. The cooperative role of S1P3 with LYVE‐1 in LMW‐HA‐induced lymphangiogenesis. Exp Cell Res. 2015;336:150‐157. 26116468 10.1016/j.yexcr.2015.06.014

[26] Moldobaeva A , Jenkins J , Zhong Q , Wagner EM . Lymphangiogenesis in rat asthma model. Angiogenesis. 2017;20:73‐84. 27787629 10.1007/s10456-016-9529-2 PMC5303156

[27] Kärpänen T , Heckman CA , Keskitalo S , et al. Functional interaction of VEGF‐C and VEGF‐D with neuropilin receptors. FASEB J. 2006;20:1462‐1472. 16816121 10.1096/fj.05-5646com

[28] Nishida‐Fukuda H , Araki R , Shudou M , et al. Ectodomain shedding of lymphatic vessel endothelial hyaluronan receptor 1 (LYVE‐1) is induced by vascular endothelial growth factor A (VEGF‐A). J Biol Chem. 2016;291:10490‐10500. 26966180 10.1074/jbc.M115.683201 PMC4865900

[29] Li J , Yi H , Liu Z , et al. Association between VEGFR‐3 expression and lymph node metastasis in non‐small‐cell lung cancer. Exp Ther Med. 2015;9:389‐394. 25574203 10.3892/etm.2014.2091 PMC4280945

