# Correction to “Fucoxanthin inhibits tumour‐related lymphangiogenesis and growth of breast cancer”

**DOI:** 10.1111/jcmm.18382

**Published:** 2024-08-26

Wang J, Ma Y, Yang J, et al. Fucoxanthin inhibits tumour‐related lymphangiogenesis and growth of breast cancer. *J Cell Mol Med*. 2019;23(3):2219‐2229.

In Wang et al.^1^ the image for Figure 5B, we mistakenly duplicated 25 μM pictures with control group, which did not affect the results of the migration experiment. The correct figure is shown below. The authors confirm all results and conclusions of this article remain unchanged.

**FIGURE 5:**
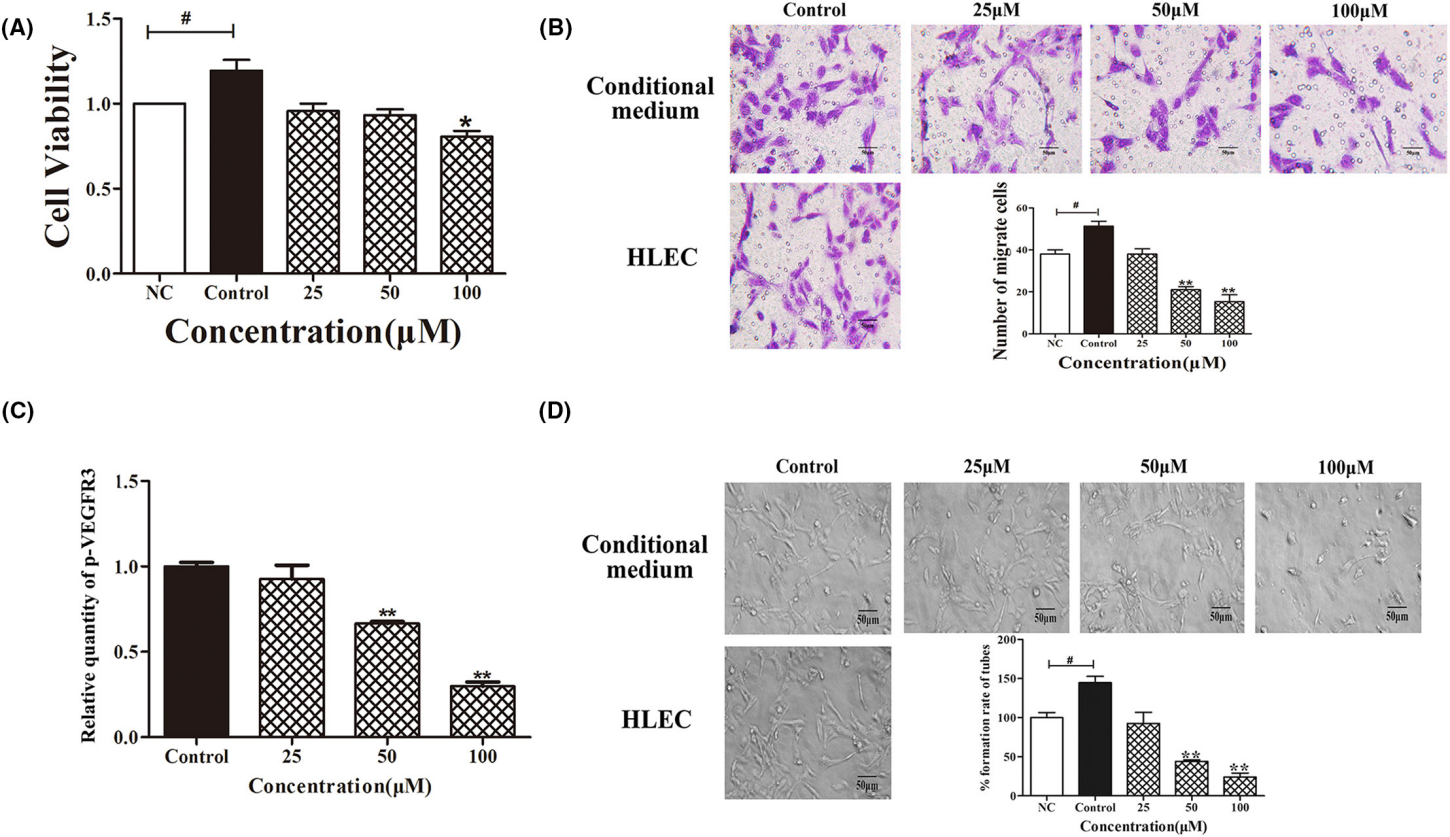
Fucoxanthin inhibits tumour‐induced lymphangiogenesis in vitro. Human lymphatic endothelial cells (HLEC) were incubated in conditional medium with or without fucoxanthin (25, 50, 100 μmol/L) for 24 h. (A) Cell viability, (B) migration and (D) tube formation are decreased in HLEC. (C) Results of ELISA showing fucoxanthin decreases phosphorylation of vascular endothelial growth factor receptor‐3. NC, negative control. Data are shown as mean ± SD; *p < 0.05 versus NC; *p < 0.05, **p < 0.01 versus controls (one‐way ANOVA).
